# Novel technique to reduce prolapsed device in atrial septal defect closure

**DOI:** 10.3389/fcvm.2023.1164061

**Published:** 2023-05-16

**Authors:** Li-Chin Liao, Sheng-Ling Jan, Ming-Chih Lin, Ho-Hsun Lee, Yun-Ching Fu

**Affiliations:** ^1^ Department of Pediatrics, Wuri Lin Shin Hospital, Taichung, Taiwan; ^2^ Department of Pediatrics, Children’s Medical Center, Taichung Veterans General Hospital, Taichung, Taiwan; ^3^ Good Day Psychiatric Clinic, Taichung, Taiwan; ^4^ Department of Pediatrics, School of Medicine, National Chung Hsing University, Taichung, Taiwan; ^5^ Department of Pediatrics and Institute of Clinical Medicine, National Yang Ming Chiao Tung University, Taipei, Taiwan

**Keywords:** atrial septal defect, push back technique, left atrial disc prolapse, transcatheter closure, transcatheter atrial septal defects closure

## Abstract

**Objective:**

Transcatheter closure of atrial septal defect (ASD) has become an alternative treatment to surgical repair. One of the challenges is the prolapse of the left atrial disc during the procedure. Many techniques have been developed to prevent the prolapse but not reduce it. In this study, we present a novel technique, termed push back technique, that help reduce the prolapsed device.

**Methods:**

We enrolled 24 patients (8 males, 16 females) between May 2008 and January 2023 who underwent the push back technique during transcatheter closure of ASD in Taichung Veterans General Hospital. We recorded the hemodynamic data, success rate and complications including device embolization/migration, valvular regurgitation, pericardial effusion, and residual shunt.

**Results:**

The median age was 6.3 years (1.2-70.5 years) and the median weight was 19.1 kg (7.8–90 kg). Fifteen (62.5%) patients had mild pulmonary hypertension. The median Qp/Qs was 2.54 (1.5–8.8). The median ASD stretched size was 21.2 mm (7.7–35.3 mm). The median device size was 22 mm (8–40 mm). The median fluoroscopy time was 14 min (5–23 min) and median procedure time was 47 min (25–78 min). The push back technique successfully reduced the prolapsed device in 21 (87.5%) patients. There was no complication in all patients.

**Conclusion:**

We present a novel push back technique that can successfully reduce the prolapsed device in 87.5% (21/24) patients without complications. It is feasible, safe and effective.

## Introduction

Transcatheter closure of atrial septal defect (ASD) has become an alternative treatment to surgical repair with a high degree of safety and efficacy (1–4). The Amplatzer septal occluder is one of most commonly used devices for ASD closure in many countries. However, it is still challenging when a patient has a large ASD with a deficient rim (5). One of the challenges is the prolapse of the left atrial (LA) disc to the right atrium (RA) during the procedure (6). Traditionally the device needs to be retrieved and redeployed, thereby increasing the fluoroscopy and procedure time, which also increases the risks of arrhythmia, perforation, and thromboembolism. Many techniques have been developed to prevent the prolapse including pulmonary vein, balloon assisted, Wahab, parallel wire techniques etc. but not reduce it (7–18). Moreover, complex maneuvers or additional equipment increase the possibility of injury to the myocardium or blood vessels (19, 20). In this study, we present a novel technique, termed push back technique, that help reduce the prolapsed device.

## Methods

**Figure 1:**
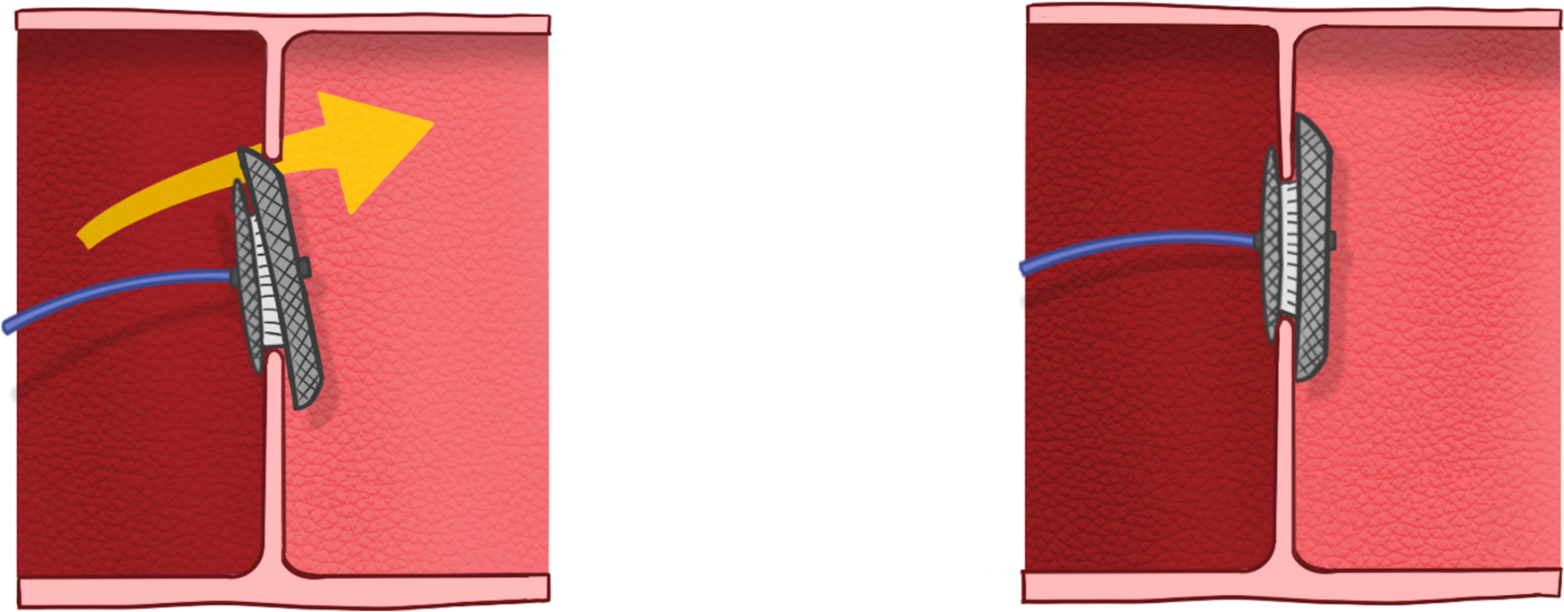
Line drawing of the push back technique.

**Figure 2:**
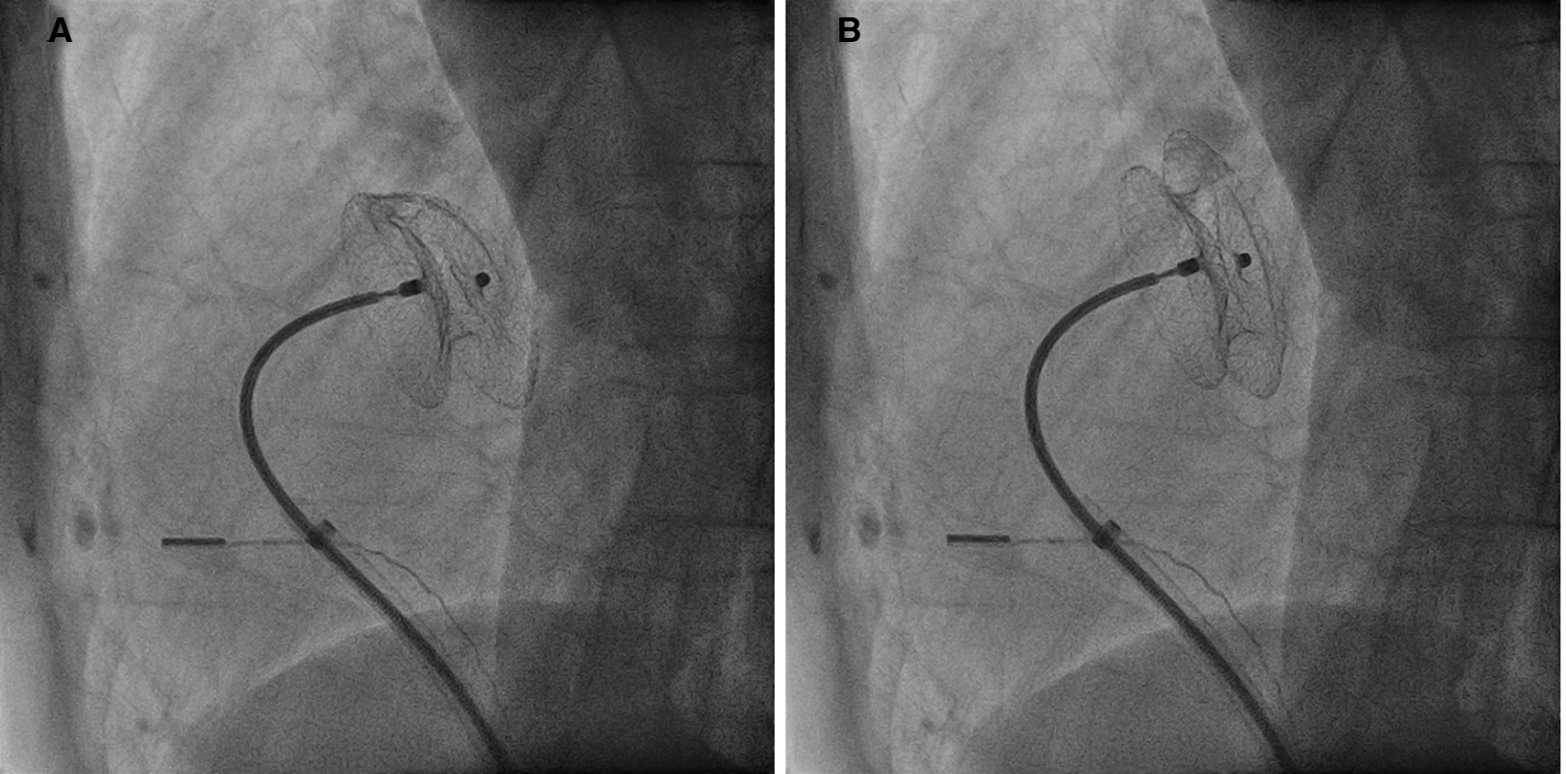
Fluoroscopy images showing the prolapsed superioanterior part of left atrial disc (**A**) which was successfully reduce (**B**) with the push back technique.

**Figure 3:**
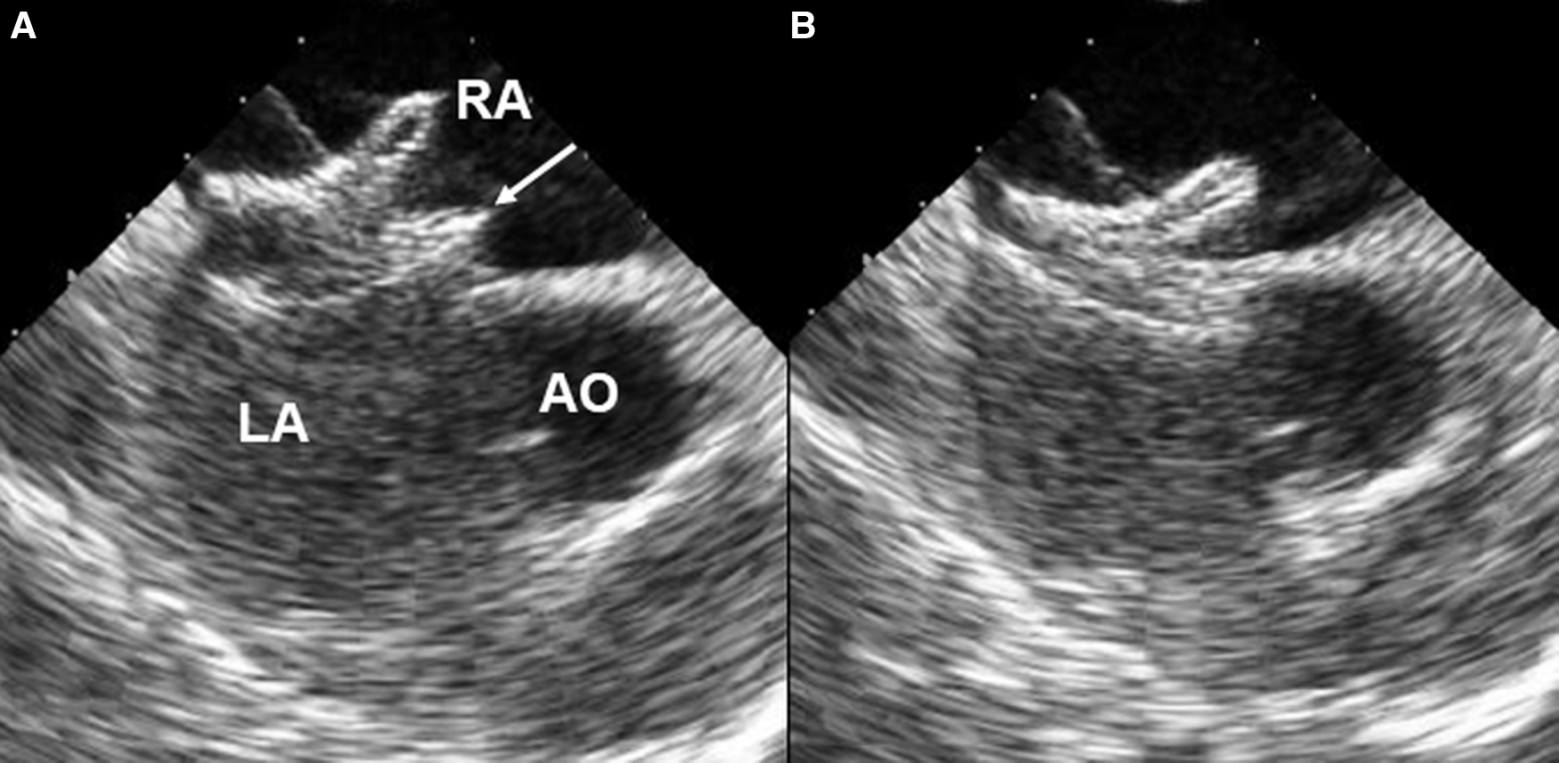
Intracardiac echocardiography showing the prolapsed superioanterior part (arrow) of left atrial disc (**A**) which was successfully reduced (**B**) with the push back technique. AO, aorta; RA, right atrium; LA, left atrium.

We enrolled 24 patients (8 males and 16 females) between May 2008 and January 2023 who underwent the push back technique during transcatheter closure of ASD in Taichung Veterans General Hospital. All patients were treated under local anesthesia and intravenous sedation without endotracheal intubation. A single dose of cefazolin and heparin (70 IU/kg) was administered to all patients at the beginning of the procedure. Vascular access was via the femoral vein in all patients. All patients right-side hemodynamic variables were monitored, with particular attention paid to pulmonary artery pressure. After the ASD stretched size was measured using an AGA sizing balloon, an appropriate device was selected. After crossing the ASD, the catheter was left in the left upper pulmonary vein with a 0.038-inch super stiff wire. Then the catheter was exchanged for a long delivery sheath. In our 24 cases, the superioanterior part of LA disc had prolapsed into the RA and needed repositioning. After RA disc deployment, we performed the push back technique using the delivery cable to bring the prolapsed LA disc back to the LA. Figure 1 showed the line drawing of the push back technique. The push back technique was performed by pulling the long sheath downwards and away from the screw of the right disc to the low RA level. Then we pushed the cable upward forcefully to make a 90-degree perpendicular leftward posterior curve (Figure 2), after which we directly pushed the device from an anterior to posterior direction (Supplementary Video S1). We closely observed the superioanterior part of the left disc. When the prolapsed LA disc was reduced, we stopped pushing the cable to avoid pushing the whole unit into the LA. After the good position of the device was confirmed by echocardiography (Figure 3), the device was released. Device embolization/migration, valvular regurgitation, pericardial effusion, and residual shunt were followed by echocardiography. Antiplatelet therapy such as acetylsalicylic acid was administered post-procedure for 6 months.

## Results

The patients' demographic data were summarized in Table 1. The median age was 6.3 years (1.2–70.5 years) and the median weight was 19.1 kg (7.8–90 kg). Fifteen (62.5%) patients had mild pulmonary hypertension. The median pulmonary to systemic flow ratio (Qp/Qs) was 2.54 (1.5–8.8). The median ASD stretched size was 21.2 mm (7.7–35.3 mm). The median device size was 22 mm (8–40 mm). The median fluoroscopy time was 14 min (5–23 min) and median procedure time was 47 min (25–78 min). After failure of standard technique, 11 (48%) patients, had tried more than one modified technique including 8 patients using left upper pulmonary vein technique and 3 patients using balloon assisted technique. One patient had 2 ASDs but received only one device closure. The push back technique successfully reduced the prolapsed device in 21 (87.5%) patients. The push back technique was performed only once in all cases. There was no complications of embolization/migration, erosion, increased valvular regurgitation or death. Residual shunt was found in 2 patients, which had reduced at the 3-months follow-up. None of the cases developed atrial fibrillation.

**Table 1:** Demographic characteristics of the patient group.

No.	Sex	Age	BW	mPAP	Qp/Qs	ASD size (mm)	Device size (mm)	Device waist (mm)	Fluoroscopy time (min)	Procedure time (min)	Success
		(yr)	(Kg)	(mmHg)							
1	F	2.2	16.0	19	2.00	17.0	17	13.1	14	60	Y
2	M	4.4	15.1	29	3.00	24.7	24	19.5	14	45	Y
3	F	38.0	51.0	23	5.50	28.0	34	28.7	15	49	Y
4	F	4.0	9.5	27	1.94	11.1	12	10.7	8	55	Y
5	M	2.5	11.5	23	1.63	17.2	17	15.2	16	78	Y
6	M	2.3	11.0	19	2.58	15.9	16	15.7	12	45	N
7	M	10.1	54.0	25	2.42	24.5	26	21.6	13	35	N
8	F	2.5	14.0	22	2.29	15.1	13	12.9	6	25	Y
9	F	70.5	48.5	18	2.30	24.4	26	22.7	23	35	Y
10	F	2.6	13.0	14	1.50	7.7	8	7.1	17	55	Y
11	F	52.2	53.0	20	2.64	17.5	16	15.9	16	56	N
12	F	22.9	51.0	21	7.80	35.3	40	34.8	5	35	Y
13	M	2.7	16.0	17	2.50	20.3	17	14.5	14	62	Y
14	M	7.1	20.0	13	2.75	23.3	24	20.4	10	55	Y
15	M	38.1	80.0	25	2.22	23.8	24	19.9	14	40	Y
16	F	19.1	56.4	21	3.63	22.0	22	18.5	11	44	Y
17	F	1.2	7.8	18	1.70	11.1	12	10.9	18	70	Y
18	F	29.9	72.0	16	8.80	30.2	30	25.0	11	35	Y
19	F	4.0	14.0	21	2.70	22.0	22	20.0	11	42	Y
20	F	6.5	25.0	22	2.00	26.4	26	23.4	22	51	Y
21	F	33.0	56.0	20	2.20	19.7	22	19.8	11	40	Y
22	M	20.1	90.0	25	2.66	22.3	24	20.9	10	33	Y
23	F	6.1	18.2	16	2.90	20.0	19	16.4	19	57	Y
24	F	3.0	13.1	20	2.70	19.6	20	17.3	18	57	Y
Median		6.3	19.1	20.5	2.54	21.2	22	19.0	14	47	
Min		1.2	7.8	13	1.5	7.7	8	7.1	5	25	
Max		70.5	90.0	29	8.8	35.3	40	34.8	23	78	

mPAP, Mean pulmonary artery pressure; F, female; M, male.

## Discussion

One of the challenges of transcatheter closure of large ASD with deficient rim is the prolapse the LA disc during the procedure. When the delivery system crosses through the ASD, the natural curvature may result in poor positioning of the LA disc, i.e., not fully parallel to the atrial septum, and thus the LA disc of the device may easily prolapse into the RA. Many techniques were developed to prevent the LA disc prolapse such as using of steerable or Hausdorff sheath, balloon assissted technique, the “Wahab technique”, the parallel wire technique, and new devices with a better orientation between the LA disc and ASD or modifying the long sheath with a side hole (8–14, 18, 21). However all the above techniques are developed to prevent and not to reduce the prolapse. Some suggest selection of an oversized ASD device to reduce the possibility of prolapse but carries potential risk for perforation, device erosion, and hemopericardium (19, 20).

In this study, we present a novel technique to push back the prolapse of the LA disk back into the LA. This push back technique was successful in 21 cases (87.5%). It is important that the push back technique be performed smoothly to keep the RA disc in the RA and only push the LA disc back into the LA. This technique can be performed by a single operator without an assistant. To our knowledge, this is the first description of this novel technique to help reposition the ASO across the ASD. This technique is especially in cases with large defect or a deficient rim. The possible reason resulting in three unsuccessful cases was that the long sheath position was not far enough from the screw of the right disc, therefore, the curvature was not perpendicular to the atrial septum resulting in failure. The push back technique is relatively simple and cost-free. We suggest using this technique first when LA disc prolapse occurs. However there are still some limitations. First, as LA disc prolapse mostly occurs in cases with a large ASD or deficient rim, the operator should be proceeding cautiously when using the push back technique to avoid pushing the whole device into the LA. Second, if the size of the ASO is too large compared with the size of the ASD, the push back technique may not work.

## Conclusion

We present a novel push back technique that can successfully reduce the prolapsed device in 87.5% (21/24) patients without complications. It is feasible, safe and effective.

## Data Availability

The original contributions presented in the study are included in the article/**Supplementary Material**, further inquiries can be directed to the corresponding author.
